# Telehealth as a catalyst for smart rural development and sustainable tourism: a feasibility case study from Agrafa, Greece

**DOI:** 10.3389/fdgth.2025.1739417

**Published:** 2026-01-09

**Authors:** Yiannis Koumpouros, Androniki Kavoura

**Affiliations:** 1 Digital Innovation in Public Health Research Lab, Department of Public and Community Health, University of West Attica, Athens, Greece; 2 Department of Business Administration, University of West Attica, Athens, Greece

**Keywords:** digital health equity, Greece, regional innovation, rural development, smart destinations, sustainable tourism, telehealth, TytoCare

## Abstract

**Introduction:**

Rural regions often face persistent healthcare access challenges due to geographic isolation, aging populations, limited infrastructure, and seasonal fluctuations in demand. These challenges not only impact resident well-being but also hinder tourism development. While research has addressed rural healthcare or development separately, limited attention has been given to the synergies between telehealth, regional revitalization, and tourism. This study investigates how telehealth can act as a catalyst for both rural development and sustainable tourism in remote settings.

**Methods:**

This pilot study introduces a telehealth framework using a portable diagnostic device integrated into a broader smart village strategy. The initiative was led by the Digital Innovation in Public Health Research Lab at the University of West Attica, in partnership with local authorities and private healthcare providers. Key components included: (a) cross-sector collaboration; (b) a custom-built web platform for monitoring effectiveness; (c) training of local personnel to assist with guided remote consultations; (d) use cases such as chronic disease monitoring, acute symptom triage for tourists, and digital nomad services; and (e) policy alignment at national and European levels.

**Results:**

Preliminary qualitative findings suggest improved healthcare accessibility for residents with chronic conditions and enhanced medical support for visitors and digital nomads. The system demonstrated feasibility even in low-connectivity environments and received positive feedback from community stakeholders.

**Discussion:**

This study contributes both theoretically and practically by advancing literature on the intersection of telehealth and rural tourism development. The framework offers a replicable model for other European rural regions seeking to enhance health equity, promote digital inclusion, and attract long-term visitors. Despite challenges—such as digital literacy, infrastructure limitations, and sustainability—the pilot illustrates the strategic potential of telehealth in underserved areas. Future research will focus on longitudinal outcomes and the policy tools needed for broader scalability.

## 1 Introduction

Rural and remote regions face persistent challenges in accessing healthcare services, which not only affect resident well-being but also limit their potential for sustainable tourism and economic development (1, 2). In September 2025, the European Union addressed health inequalities in the European Union caused by obstacles to health such as unequal access to the resources that help people stay healthy based on the latest comparable data from across Europe (3). Some countries were not included in the report since countries in Central and Eastern Europe fall behind in healthcare. Furthermore, it has been reported that differences in procedures and processes also exist particularly in countries in Central and Eastern Europe, such as delays, limited access to clinical trials, and restricted availability of specialized care (4). The issue becomes even more acute for specific remote areas in Greece. In areas with aging populations and limited medical infrastructure, such as Agrafa in central Greece, these barriers are particularly acute (5). Agrafa qualifies as a “predominantly rural remote area” per Eurostat 2025 territorial typology (very low population density <50 inh/km^2^, >60 min travel to hospitals/specialists) and GRANULAR framework, exemplifying EU rural health disparities (6, 7). Although the U.S. Rural–Urban Continuum Codes (RUCC) are frequently used in North American rural health research, they are not applicable to European contexts. If compared conceptually, Agrafa would correspond to the most rural RUCC categories (8, 9) due to its extremely low population density, absence of urban centers, and long travel times to essential health services. However, in Europe the official designations follow Eurostat's 2025 territorial typology and the EU GRANULAR framework, which constitute the recognized basis for defining rurality in the Greek and EU context and under which Agrafa is classified as a “predominantly rural, remote area.” The COVID-19 pandemic further exposed and intensified these vulnerabilities, reshaping public expectations around health security and digital readiness in travel destinations (1, 8).

Telehealth has emerged as a transformative solution to these challenges, offering remote consultations, diagnostics, and monitoring that reduce the need for travel and expand access to care. Studies have shown that digital health interventions in rural settings improve chronic disease management, enhance patient outcomes, and reduce healthcare costs through preventive care models (9–11). Moreover, the pandemic accelerated the adoption of telemedicine technologies, prompting rural communities and tourism stakeholders to invest in digital infrastructure and services to meet evolving visitor expectations (6, 8). Recent studies confirm telehealth's critical role in addressing healthcare disparities in rural areas by improving access to specialty care and chronic disease management (12, 13). However, these interventions require infrastructure investment and policy support to overcome persistent financial and technological barriers (14).

This shift aligns with broader European strategies such as the Smart Villages initiative, which promotes digital inclusion and innovation-driven resilience in underserved territories (2). Despite the rapid evolution of telehealth technologies, few studies have explored their role in catalyzing smart rural development or their dual function as both a healthcare intervention and a tourism enabler. Within this context, portable telemedicine solutions like TytoCare, an FDA-cleared device enabling virtual physical exams, offers scalable, community-based interventions that support both resident health and visitor confidence (15, 16). This study presents a pilot implementation of TytoCare in Agrafa, integrated with a web-based platform developed by the Digital Innovation in Public Health Research Lab at the University of West Attica. The initiative explores whether telehealth can serve as a strategic lever for rural revitalization by improving healthcare access, supporting sustainable tourism, and fostering digital equity. It evaluates the feasibility, acceptability, and anticipated socioeconomic impact of tele-care deployment in a geographically isolated region, contributing to the growing literature on digital health as a catalyst for inclusive rural development (17).

In summary, this research positions telehealth not merely as a healthcare innovation but as a strategic lever for rural development and tourism revitalization. By systematically assessing the Agrafa pilot, the study aims to provide actionable insights for policymakers, tourism stakeholders, and healthcare providers seeking to harness digital health for the benefit of rural communities and the visitors they hope to attract. The findings will contribute to a growing body of literature on the intersection of digital health, smart tourism, and rural resilience, highlighting pathways for inclusive and sustainable growth in the post-pandemic era (1, 2, 5, 11, 16). This paper specifically emphasizes the role of cross-sector synergies—between public institutions, academic research, private healthcare, and community actors—in designing an integrative model that leverages telehealth for both healthcare delivery and sustainable regional development. The initiative is grounded in a “Smart Village” strategy that views digital health not solely as a clinical solution, but as a developmental tool with spillover effects on tourism appeal, demographic stability, and community resilience. As such, it explores a multidimensional framework where healthcare infrastructure, local governance, and tourism development are co-designed to generate mutually reinforcing benefits.

## 2 Literature review: telehealth, rural innovation, and health tourism

### 2.1 Telehealth in rural contexts

Telehealth has increasingly been framed as a strategic response to rural healthcare disparities, particularly where geography, demography, and workforce shortages intersect (18). Greece exemplifies this challenge, with rural regions like Agrafa experiencing persistent deficits in general practitioners and specialists. During tourist seasons, the healthcare burden further intensifies due to population surges and increased accident or chronic care needs (19). Telemedicine is proposed as a cost-effective, scalable solution for remote triage, chronic disease management, and emergency support (20). However, implementation success varies significantly based on local infrastructure, digital literacy, and trust in technology (21). Regions with aging populations often face resistance to unfamiliar medical technologies, despite their clinical efficacy. As such, successful adoption of telehealth tools is closely tied not just to technological readiness, but to community engagement, education, and culturally appropriate rollout strategies. TytoCare represents a new class of consumer-friendly medical technology that enables remote physical examinations typically limited to in-person clinical visits. The device integrates a camera, otoscope, stethoscope, and thermometer into a compact unit that transmits data to licensed physicians via cloud platforms. This design has allowed it to be used effectively in schools, remote clinics, and homes worldwide. Critically, the platform doesn't just improve access—it also builds confidence in remote diagnostics by ensuring physicians maintain visual and diagnostic control. This hybrid model, where laypeople collect data but doctors maintain interpretive authority, helps alleviate liability and quality concerns. Devices like TytoCare can “extend the reach of doctors into communities without requiring their permanent presence,” an especially potent concept in areas like Agrafa, where medical professionals are scarce (18).

### 2.2 Digital health, rural economies, and the rise of digital nomadism

There is growing recognition that telemedicine infrastructure can catalyze wider socio-economic gains. Beyond direct healthcare benefits, digital health systems can anchor new models of regional development rooted in wellness, remote work, and lifestyle migration. As traditional tourism evolves into longer, purpose-driven stays—especially among digital nomads and remote workers—regions that offer both natural beauty and essential digital infrastructure are emerging as competitive destinations (22). Digital nomads, defined as professionals who use telecommunications technologies to work remotely while traveling or living in diverse locations, increasingly seek destinations that blend affordability, authenticity, connectivity, and safety. While Wi-Fi and coworking spaces are often seen as baseline requirements, access to dependable healthcare—especially in isolated areas—is an underrated yet critical factor in destination choice (23). The presence of telehealth services like the one proposed in Agrafa not only supports local populations but directly aligns with the expectations of this new class of mobile professionals. Moreover, national strategies across Europe—including Greece's own digital nomad visa initiatives—are designed to attract longer-term residents who contribute economically while working for companies abroad. However, for rural destinations to capitalize on this trend, they must ensure not only digital connectivity, but also medical security—an area where telemedicine serves as both a public good and a marketing tool. By offering remote diagnostics in a region historically deprived of health infrastructure, Agrafa can position itself as a “resilient retreat” for health-conscious nomads and lifestyle migrants. The synergy between eHealth and digital nomadism also aligns with the EU's broader Smart Villages agenda, which promotes digital inclusion, sustainable growth, and innovation-driven resilience in rural territories (24, 25). In this context, the proposed telehealth solution is more than a health device—it becomes part of a strategic infrastructure enabling demographic revitalization and rural rebranding. In this context, telehealth evolves beyond a care delivery mechanism and becomes part of a broader territorial strategy. It reinforces Agrafa's identity as a resilient, future-ready rural destination capable of hosting health-conscious tourists and remote workers, thereby contributing to place branding and rural regeneration agendas.

### 2.3 Evaluation frameworks and anticipated challenges

Despite accelerated telehealth adoption in recent years, sustainable implementation in rural areas requires overcoming infrastructure, policy, and patient engagement challenges (26, 27). Although the telehealth pilot in Agrafa is still in early stages, lessons from similar initiatives suggest key dimensions for evaluating its success:

• Access and equity: Reduction in travel needs, especially for vulnerable residents
• Continuity of care: Integration with existing health systems and local providers
• Patient experience: Ease of use, satisfaction, and trust in remote diagnosis
• Economic value: Savings in emergency transport and increased tourist confidence

However, challenges remain. These include limitations in internet coverage, training requirements for device operators, and public trust in non-physical consultations. These factors have been proactively addressed in the design and monitoring of the Agrafa implementation.

## 3 The telehealth pilot in Agrafa: implementation plan and strategic outlook

This study adopts a feasibility and implementation case study design to evaluate the deployment of a telehealth system in a remote rural context. The Agrafa region, located in the mountainous hinterland of central Greece, is emblematic of the challenges faced by remote rural territories. Based on European rural typologies, including Eurostat's classifications and recent GRANULAR analyses, such regions are characterized by very low population density, long travel times to essential services, and persistent structural limitations in service provision — all factors that make them highly relevant contexts for digital health deployment (6, 7). Its population is characterized by demographic aging, declining birth rates, and sparse settlement patterns. Access to primary healthcare is limited by both geographic isolation and systemic workforce shortages. Most residents must travel over an hour to reach the nearest general practitioner, while seasonal population surges during tourist months further burden the already limited health infrastructure. These conditions identified Agrafa as a strategic setting for piloting a telehealth intervention that is scalable, rapidly deployable, and does not rely on permanent clinical infrastructure. The pilot was designed from inception as a model of integrated rural development, grounded in both healthcare and tourism policy objectives. Special attention was given to aligning local governance (municipality of Agrafa), private healthcare services (EmergencyHelp), and academic research (University of West Attica). To this end, the pilot project was co-designed by the Digital Innovation in Public Health (DigInHealth) Research Lab at the University of West Attica, in collaboration with the Municipality of Agrafa, EmergencyHelp (a private medical service provider and TytoCare's distributor in Greece), and a local community center acting as a health access hub. The initiative aimed to install telehealth equipment at selected village locations and to train community personnel—such as social workers or support staff—to facilitate guided remote consultations. Physicians based in Athens were connected via a secure web portal, enabling live consultations and follow-up support. Training was delivered through a combination of blended workshops and device-based simulations, with particular attention to the needs of:

• Elderly users and informal caregivers
• Digitally inexperienced or low-literacy individuals
• Emergency care triage protocols

The implementation plan included:

• Installation of one telehealth unit in the village of Granitsa, located in a refurbished municipal facility
• A secure cloud-based data infrastructure, compliant with GDPR and national privacy regulations
• A custom-built web platform developed by the DigInHealth Lab to monitor system performance and collect user data

**Figure 1:**
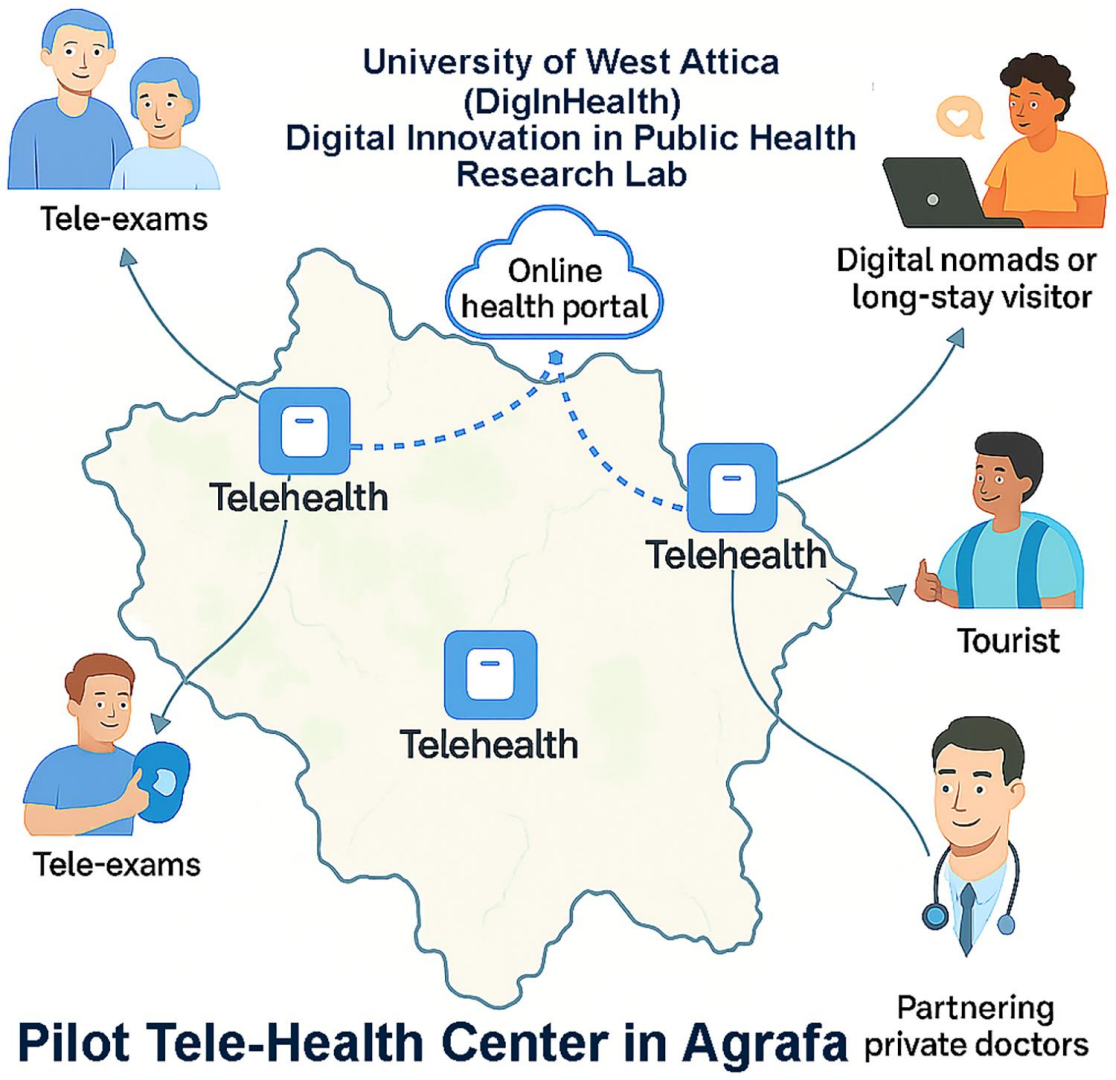
Telehealth unit installed in a municipal facility in Granitsa, Agrafa.

The system operates using a 4G uplink and requires only standard broadband connections. Figure 1 shows the installed unit in operation in Agrafa. A critical component of the implementation was the involvement of community-based personnel who were trained to act as facilitators during guided consultations. This addressed both digital exclusion and built local capacity for sustained operation. The emphasis on training non-specialist actors, such as municipal workers and social care personnel, reflects a scalable strategy for rural areas where formal health professionals are scarce.

### 3.1 Study design and evaluation approach

This project follows a feasibility and implementation case study design to assess the early deployment and perceived impact of a telehealth solution in the remote rural region of Agrafa, Greece. The approach prioritizes practical insight into real-world implementation rather than hypothesis-driven clinical outcomes.

The evaluation focuses on four core feasibility domains:

• Acceptability: User satisfaction and willingness to engage with the platform, based on informal interviews and community reports.
• Adoption: Stakeholder engagement, training outcomes, and local facilitator involvement.
• Practicality: Infrastructure compatibility, digital literacy needs, and system operability under real-world constraints.
• Preliminary Reach: Initial frequency of platform use by residents and visitors, where data is available.

Stakeholders include local municipal leaders, public health staff, community volunteers, and residents. Informal stakeholder feedback was collected during training sessions and after initial use, and is presented thematically. No formal patient records were collected at this stage, and data is anonymized and qualitative in nature. The implementation aligns with WHO's Digital Health Intervention Evaluation Framework and the RE-AIM model for implementation science.

To collect early-stage feedback, informal interviews (*n* = 6) and structured questionnaires were conducted with a small but strategically selected group of local stakeholders: one hotel owner, one restaurant manager, one municipal employee, one permanent resident, one hotel employee, and one local service provider. The municipal employee was included because he serves as a year-round liaison for local services and frequently assists residents and visitors in accessing community resources. His institutional role, continuous presence, and direct interactions with vulnerable residents made him an appropriate facilitator despite not belonging to the other occupational categories represented. A general practitioner who periodically serves the village under a fixed-term municipal contract was also trained in the use of the telehealth system so that she could request remote specialist second opinions from physicians when needed. Although involved clinically, she did not form part of the six trained community facilitators. For clarity, the evaluation distinguishes three participant groups: (a) the six trained community facilitators (non-clinical, device guidance); (b) 24 residents and 6 tourists who used the system for teleconsultations; and (c) one local general practitioner, whose role was limited to seeking specialist second opinions rather than facilitating device operation. These six individuals were not only early users but also identified as key facilitators of the service. The hotel and restaurant owners were chosen due to their year-round presence in the area and their locations at critical access points for tourists. Their role was envisioned as twofold: to assist older or digitally inexperienced residents in using the telehealth unit and to inform and guide visitors and tourists regarding the availability of the service. Their digital literacy levels were sufficient to allow rapid onboarding and effective operation of the platform after basic training. Data were collected after training sessions and initial system use. Although the sample was small, it provided valuable insights into user experience, perceived benefits, and implementation barriers. The findings were summarized thematically by the project team without formal coding and should be considered preliminary. Qualitative inputs from interviews and field observations were thematically grouped into recurring categories related to usability, confidence, and operational challenges to ensure a minimum level of analytical structure (see Table 1).

**Table 1:** Thematic grouping of stakeholder interview findings (*n* = 6).

Theme	Description	Representative Quote
Facilitator confidence	Rapid increase in self-efficacy following training	“After two sessions, I felt confident guiding residents.”
Usability & perceived safety	Device viewed as simple and reassuring for vulnerable users	“I feel safer now — I don't need to travel far to see a doctor.”
Digital literacy barriers	Older adults required more assistance and repetition	“Some older visitors hesitate, but return after the first experience.”

These 30 cases represent end-users of the service and are analytically distinct from both the trained facilitators and the GP, whose involvement related exclusively to clinical guidance rather than system operation. A more comprehensive evaluation phase with larger samples is planned during the forthcoming 12-month monitoring cycle.

This pilot was conducted as a real-world service implementation project and not classified as human-subject research. Nonetheless, ethical safeguards were integrated throughout the process. All participants involved in interviews or feedback provision signed a consent form prior to participation, and no personally identifiable data were collected—only anonymized testimonials were used. The telehealth platform itself is both FDA-approved and fully compliant with the General Data Protection Regulation (GDPR). Before each teleconsultation, users were informed directly through the platform interface about the handling of their sensitive health data and asked to provide digital consent in accordance with GDPR principles. Additionally, an online questionnaire was provided post-session, in which users gave explicit consent for their anonymized responses to be used for service evaluation and research dissemination. All communication was encrypted, and no personal data and health records were stored locally or permanently during this feasibility phase. Furthermore, formal approval was obtained from the Municipality of Agrafa to administer the anonymous questionnaires and conduct interviews with participating residents, as part of the local service quality improvement strategy.

### 3.2 Anticipated impact and use cases

As part of the pilot's initial operational phase, 30 real-time teleconsultations were completed using the deployed unit in Granitsa. Of the 30 teleconsultations, 24 involved permanent residents and 6 involved tourists or short-term visitors, allowing for a clearer distinction between local healthcare needs and tourism-related cases. Table 2 provides a descriptive summary of the 30 teleconsultations conducted during the pilot, broken down by resident vs. tourist status and by clinical use-case category.

**Table 2:** Descriptive breakdown of the 30 teleconsultations conducted during the pilot phase.

Category	Residents (*n* = 24)	Tourists (*n* = 6)	Total	Age range	Main complaints
Chronic Follow-up	12	0	12	55–82	Hypertension, diabetes, COPD
e-Prescription	8	2	10	45–75	Medication renewal
Symptom Reporting	3	3	6	28–65	Fever, ear pain, cough
Acute Triage	1	1	2	35–60	Minor injury assessment

The 6 tourist cases represent all unique individuals, each corresponding to one distinct teleconsultation.

These descriptive findings correspond to the “Reach” and “Adoption” dimensions of the RE-AIM framework, illustrating early uptake among both resident and visitor populations. These involved residents and tourists who presented with minor acute symptoms or chronic follow-up needs. Connectivity was ensured via a dedicated 4G SIM card and router provided by the implementation team, which bypassed local broadband limitations and guaranteed stable communication. Table 3 summarizes the preliminary feasibility indicators of the Agrafa pilot, mapped onto the core dimensions of the RE-AIM framework to clarify early uptake, operational viability, and user experience.

**Table 3:** Preliminary feasibility indicators of the Agrafa telehealth pilot mapped to the RE-AIM framework domains.

RE-AIM dimension	Indicators (pilot data)	Findings
Reach	30 consultations (24 residents, 6 tourists)	Early uptake despite low-connectivity
Effectiveness	Satisfaction quotes/themes	"safer, no long trips”; usability positive
Adoption	6 facilitators trained (60 min, 4 scenarios)	Confidence gains post-training
Implementation	4G router; GDPR-compliant platform	Feasible; barriers addressed thematically
Maintenance	Planned 12mo tracking; municipal support	Ongoing tracking secured

Qualitative Themes from 6 Stakeholder Interviews (Thematic Analysis):

1. Increased Confidence (RE-AIM: Adoption) – “Now I can guide anyone confidently after training” (hotel owner).
2. Usability Strengths (RE-AIM: Implementation) – “Simple for tourists, 4G works reliably even in remote areas” (restaurant manager).
3. Digital Literacy Barriers (RE-AIM: Reach) – “Elderly residents need more practice time” (municipal employee).

These findings directly correspond to RE-AIM indicators — including “Reach” (resident vs. tourist uptake), “Adoption” (facilitator onboarding), and “Implementation” (operability via 4G). They also map to the WHO Digital Health Monitoring and Evaluation Framework, particularly the domains of Acceptability, Practicality, and Feasibility. To support these consultations, six local stakeholders (hotel owner, restaurant manager, municipal employee, permanent resident, hotel employee, local service provider) were selected and trained to serve as community-based facilitators. In parallel, the general practitioner assigned to the area received technical training on the system to request clinical clarifications and to obtain remote specialist second opinions, but he was not included among the six trained facilitators. Each participant received one-on-one training sessions lasting approximately 60 min, during which they practiced four representative use-case scenarios to understand the device's full functionality. Their role was to assist elderly residents or unfamiliar users and guide tourists in accessing the telehealth platform effectively.

While these results are preliminary and based on early deployment, they confirm the technical and operational feasibility of the system under real-world conditions. A structured 12-month evaluation phase is planned to capture broader usage patterns and assess long-term impacts.

As part of the implementation case study approach, while quantitative data is yet to be collected, early modeling and qualitative stakeholder feedback suggest three main use cases with high projected impact:

Primary Use Cases:

1. Chronic Disease Monitoring
  Residents living with hypertension, diabetes, or chronic respiratory conditions (e.g., COPD- Chronic Obstructive Pulmonary Disease) can monitor vital parameters and receive guidance from physicians based in urban centers. This is expected to reduce unnecessary emergency department visits and enable preventive care.
2. Acute Symptom Triage for Tourists
  During the high season, local tourism operators and hospitality venues refer visitors experiencing minor symptoms (e.g., fever, cough, ear pain) to the telehealth unit. This is expected to enhance Agrafa's positioning as a health-secure rural destination. In fact, preliminary observations from the pilot indicate that 6 out of the 30 initial teleconsultations were initiated by tourists, suggesting early engagement from visitors.
3. Support for Digital Nomads and Long-Stay Remote Workers
  With rising interest in rural locations as work-from-anywhere bases, reliable access to medical services has become a differentiating factor. The availability of telehealth in Agrafa aligns with emerging research showing that healthcare access influences destination choices among digital nomads (23, 28). This suggests that digital health infrastructure may not only address clinical access gaps, but also becomes a strategic development asset—linking health security with tourism branding and local economic renewal. These implications should be interpreted as forward-looking projections rather than established outcomes, as the pilot did not collect tourism or economic performance metrics.

Additional Anticipated Impacts:

• Rural Development Synergies

The design of the Agrafa pilot reflects intentional alignment with rural development goals. By embedding telehealth within community spaces and engaging local actors in its operation, the project contributes to employment, skills development, and the broader social economy. These ripple effects support endogenous development models where digital innovation fosters local empowerment rather than dependency on external actors. Additionally, the initiative contributes to the region's digital profile, attracting future investment in smart infrastructure, tourism services, and health innovation clusters.

• Platform Monitoring and Policy Interface

**Figure 2:**
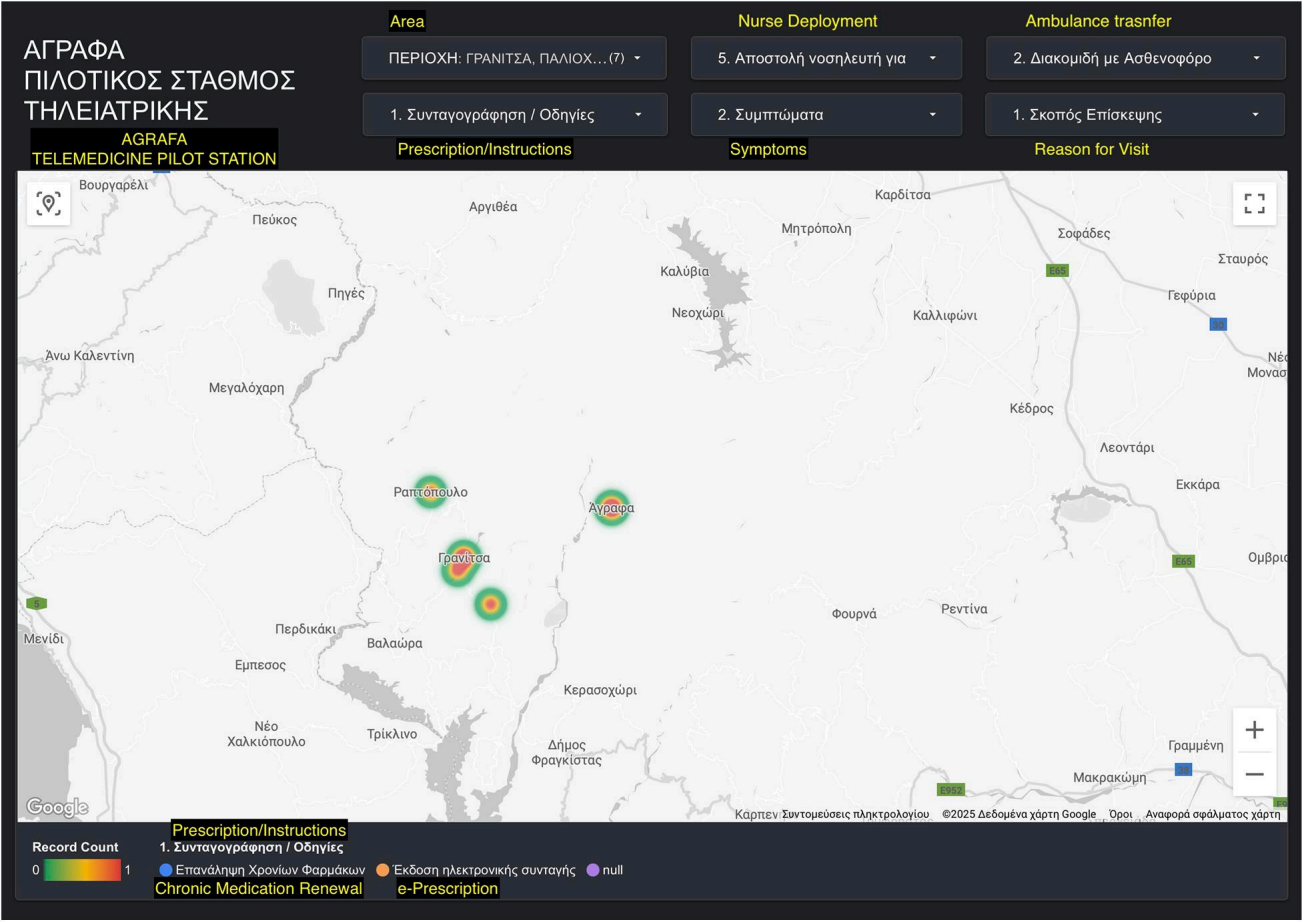
Interactive web-based platform developed by the DigInHealth Research Lab to monitor telehealth usage and support real-time data visualization. The platform maps teleconsultation activity across multiple villages, categorizing visits by type (e.g., electronic prescription issuance, chronic medication renewal), and provides real-time, filterable visualizations for decision-making support. Screenshot from: https://lookerstudio.google. com/reporting/3a1be866-7719-4166-98fa-47ddcfff309a/page/FF5QF.

The custom-built web platform developed by the DigInHealth Research Lab served a dual role: it enabled remote consultations and supported ongoing monitoring of system usage and community engagement (Figure 2). Designed specifically for the pilot, the platform collected both usage metrics and qualitative feedback to assess consultation effectiveness, identify service trends, and inform local decision-making.

An interactive, map-based dashboard displays data in real time across participating villages, using heat map visualizations to illustrate the density and distribution of telehealth interactions. Case categories—such as electronic prescription requests, chronic disease follow-up, symptom reporting, and emergency service coordination—can be filtered by variables like visit purpose, presenting symptoms, or action taken (e.g., prescription renewal, nurse dispatch).

This geographically disaggregated view provides actionable insights for local health authorities and research teams, supporting responsive service planning and contributing to the evaluation of pilot scalability. Additionally, the platform aligns with national digital health strategies and EU Smart Villages objectives by enhancing transparency and enabling data-driven policy development.

Early qualitative feedback from community members indicates a positive reception of the telehealth service. One elderly resident noted, “*I feel safer and no longer worry about long trips to the city doctor—now the help is here.”* A local facilitator added, “*We see people who were hesitant at first, but after the first visit, they bring their neighbors along.”* Such narratives underscore the importance of perceived convenience and local trust in the successful adoption of digital health interventions in rural communities. Across the qualitative material, three dominant themes emerged: (a) increased facilitator confidence, (b) device usability strengths and limitations, and (c) contextual barriers such as digital literacy among older adults.

### 3.3 Evaluation framework

To assess feasibility and inform scalability, the following indicators will be tracked over a 12-month period, following evaluation dimensions adapted from the WHO Digital Health Monitoring and Evaluation Framework and the RE-AIM model (Reach, Effectiveness, Adoption, Implementation, and Maintenance) (29, 30):

• Number of completed remote consultations per month (Reach, Effectiveness)
• User satisfaction (via surveys) (Adoption)
• Reduction in health-related travel to Karpenisi, Lamia or other urban centers (Effectiveness)
• Tourist feedback (collected through local hostels and guesthouses) (Reach, Acceptability)

Where possible, indicators will be benchmarked against similar pilots in comparable EU settings.

Long-term, this pilot may serve as a replicable model for other mountainous or island regions in Greece, potentially informing national Smart Villages strategies and EU-funded digital health programs. In keeping with the feasibility study design, this evaluation framework will guide both formative (process) and summative (outcome) insights to inform potential scale-up scenarios.

### 3.4 Training and capacity building

Community personnel, including municipal staff and social service coordinators, were trained through blended workshops combining device handling, scenario-based simulations, and digital health literacy modules. The training focused on ensuring guided use of the TytoCare device by older residents, emergency scenarios for tourists, and onboarding digital nomads unfamiliar with the Greek healthcare system. A user manual and support line were also developed for sustained use beyond the pilot phase. The involvement of community actors in training sessions emphasized the democratization of digital health tools and the empowerment of non-medical personnel to serve as digital health intermediaries—a key factor in reducing digital exclusion and fostering community resilience.

## 4 Expected advantages and policy implications

From its inception, the pilot was conceptualized as a platform for systemic integration—bringing together regional governance, healthcare innovation, and smart destination development into a single cohesive model. The implementation of Telehealth in Agrafa is designed not only to address immediate healthcare gaps but also to generate long-term value across three strategic axes: improved health equity, tourism enhancement, and rural resilience. Although outcome data from the pilot are still forthcoming, analogous deployments and theoretical frameworks suggest several foreseeable advantages. These expected outcomes form the basis for future policy alignment at local, national, and European levels. This further reinforces the conceptualization of health infrastructure not merely as a cost center, but as an enabling factor for sustainable development—enhancing resilience, digital inclusion, and territorial attractiveness in line with Smart Villages principles.

### 4.1 Health system benefits: access, equity, and efficiency

One of the most direct impacts of the telehealth pilot is its contribution to health equity in isolated populations. By decentralizing diagnostics and allowing for remote consultations, residents—especially elderly or mobility-limited individuals—gain timely access to clinical-grade examinations without the burden of long-distance travel. This is particularly significant for patients with chronic conditions such as hypertension, COPD, and diabetes, where continuous monitoring is essential for preventing acute episodes (31, 32). Furthermore, telehealth helps optimize regional healthcare resources. Physicians in urban centers such as Lamia or Athens can provide remote assessments, thereby increasing efficiency and allowing specialists to manage rural patient loads asynchronously. The integration of patient data into centralized health registries enhances continuity of care and facilitates early intervention protocols. In emergency contexts—e.g., winter weather disruptions, natural disasters, or road closures—telehealth provides a resilience layer, ensuring clinical triage remains possible when physical access is compromised (24). These observations reflect initial feasibility outcomes drawn from local implementation experience rather than quantitative impact data, which is currently being collected. The thematic patterns identified in the qualitative findings strengthen this interpretation by illustrating the early dynamics of user acceptance, operational practicality, and facilitator readiness. Maintaining this distinction between facilitators, end-users, and the GP is essential for accurately interpreting feasibility outcomes and understanding the specific functional expectations of each group.

### 4.2 Tourism competitiveness and safety branding

Health infrastructure is an *often-overlooked pillar* of tourism attractiveness in remote areas. For destinations like Agrafa, known for hiking, eco-tourism, and seasonal festivals, the availability of digital diagnostics via telehealth may in the future reinforce the destination's perceived health assurance and emergency readiness, contributing to a broader 'Safe Travel' framing. Visitors with preexisting health concerns (e.g., asthma, heart disease, immunosuppression) may be more inclined to travel to rural destinations that offer on-demand medical access, even if not physically located near a hospital (33). This is aligned with recent health tourism literature, which emphasizes perceived medical preparedness as a decisive variable in rural travel choice (23). The pilot also supports Greece's digital nomad visa program, offering extended-stay professionals not only Wi-Fi and natural beauty but also modern medical infrastructure. As digital nomads often stay for weeks or months, particularly in nature-rich environments, telehealth access becomes a prerequisite for inclusion in this mobility trend (28, 34). It is important to note that these tourism- and digital-nomad–related implications remain hypothetical at this stage, as the current feasibility study did not include measurement of actual travel behavior, economic indicators, or destination competitiveness.

### 4.3 Rural revitalization and smart village strategy

At a structural level, the telehealth pilot is consistent with EU Smart Villages initiatives, which promote the use of digital tools to revitalize rural economies through social innovation, inclusive services, and cross-sector integration (24). This is in line with broader trends in rural innovation, where transport, digital infrastructure, and healthcare are seen as interconnected pillars of regional sustainability (35). By anchoring the pilot within local health and tourism networks, Agrafa is positioning itself as a testbed for rural digital transformation. Beyond health and tourism, the existence of such a system may catalyze additional socioeconomic benefits, including:

• Increased confidence among potential returnees or retirees who may consider relocating from urban centers.
• Interest from private sector partners, such as rural co-working hubs or eco-lodges.
• Enhanced eligibility for EU development funds, which prioritize digital health solutions in cohesion policy frameworks.

### 4.4 Policy and scalability recommendations

As this pilot is positioned as a feasibility and implementation case study, policy recommendations emerging from it are preliminary but grounded in real-world deployment experience. To leverage the full potential of the telehealth pilot, several policy measures are advisable:

1. Formalize cross-sector coordination between health authorities and tourism boards.
2. Subsidize broadband infrastructure and mobile health equipment in remote areas as part of public health investment.
3. Integrate telehealth into national emergency preparedness plans, particularly in geographically isolated rural regions.
4. Develop metrics for telehealth's contribution to rural economic activity, especially where tourism and long-stay digital residency intersect.

**Figure 3:**
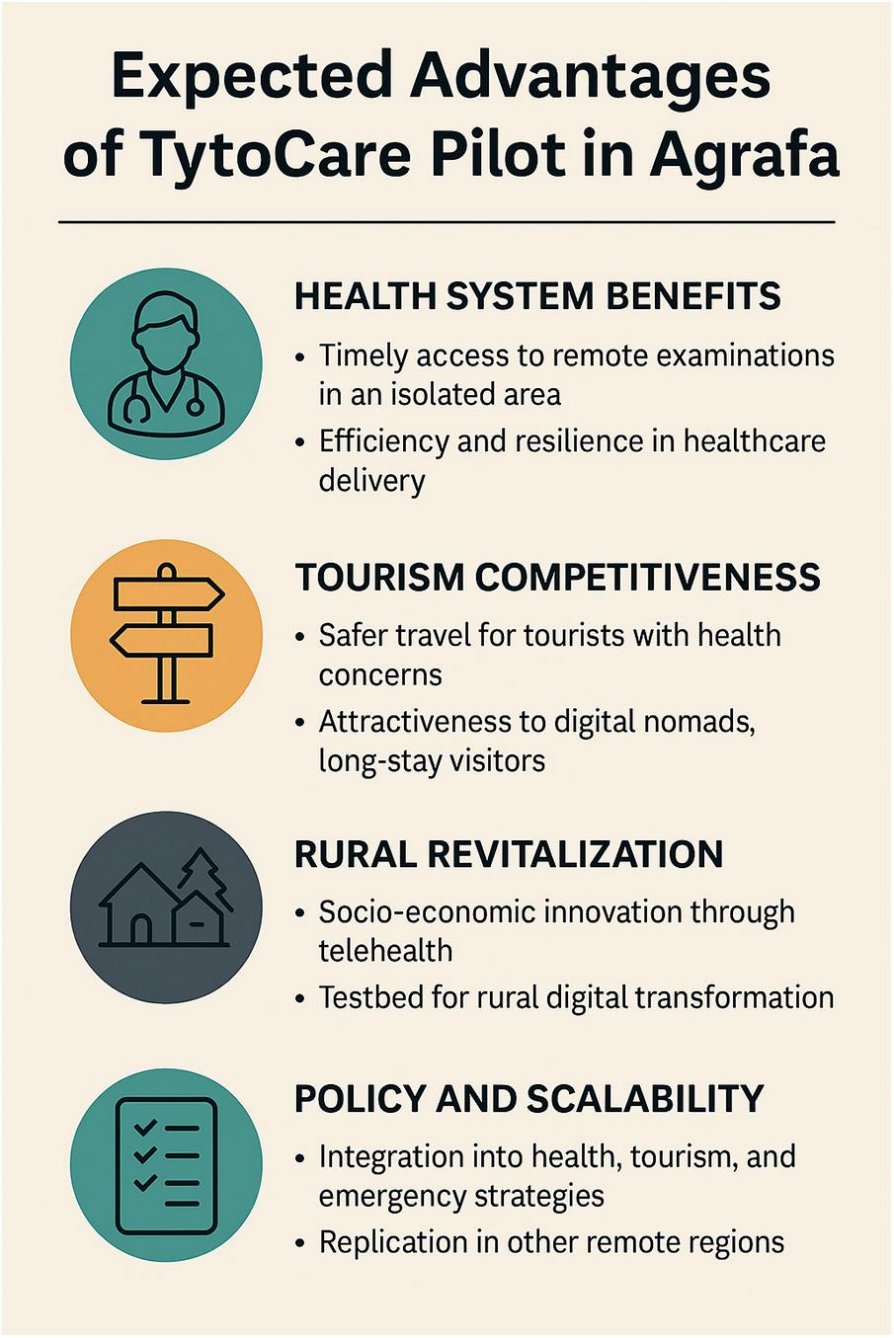
Policy infographic.

These recommendations align with the European Commission's Digital Decade targets and WHO's digital health guidelines, which emphasize cross-sector integration, digital inclusion, and health equity in underserved areas (29, 30). Scalability depends on documenting the pilot's operational workflows, collecting stakeholder feedback, and ensuring interoperability with Greece's broader health information systems. Regions with similar geographic and demographic profiles—such as Zagori, Evrytania, and parts of Crete—could benefit from similar deployments. A visual policy roadmap is presented in Figure 3, summarizing the strategic pillars for replication and integration. Furthermore, the pilot served as a vehicle for local-to-national knowledge transfer, with policy briefs submitted to the Ministry of Health and the Ministry of Tourism. These included recommendations for integrated rural telehealth funding, inclusion of tourism-specific indicators in telemedicine KPIs, and alignment with European Commission Smart Villages directives. Additionally, the project serves as a vehicle for promoting policy dialogues at the intersection of rural innovation, digital transformation, and public health inclusion. Its strategic alignment with European initiatives such as the Digital Decade and Smart Villages enables multi-level policy synergies. To ensure scalable and sustainable telehealth services, targeted policy interventions focusing on broadband expansion, training, and reimbursement reforms are essential (36). Collaborative efforts between local health services and governments can improve digital health equity outcomes.

## 5 Challenges and limitations

Despite the promising goals of the telehealth pilot in Agrafa, several interrelated challenges may affect both the effectiveness and long-term scalability of the Agrafa telehealth pilot. These are consistent with global barriers to rural telemedicine adoption and span infrastructure, digital literacy, workforce integration, legal compliance, and financial sustainability (see Table 4).

### 5.1 Infrastructure and connectivity constraints

One of the most fundamental limitations is intermittent digital infrastructure, which affects both connectivity and device functionality. Many remote areas in Greece, including parts of Agrafa, still suffer from low broadband penetration, unstable mobile coverage, and outdated telecommunications hardware. These conditions are known to obstruct telehealth implementation in rural environments worldwide (37). Without investment in stable broadband or mobile networks, devices such as TytoCare cannot reliably function in home settings, necessitating the use of centralized community access hubs, which, while functional, reduce the convenience and scalability of truly decentralized telecare (38).

**Table 4:** Summary of key challenges identified in the Agrafa telehealth pilot and proposed solutions based on implementation experience and relevant literature.

Category	Challenge	Proposed solution
Infrastructure	Weak broadband, lack of reliable mobile network	Use centralized access hubs; advocate for subsidized broadband investment (48)
Digital Literacy	Low digital confidence among older adults and new users	Offer ongoing digital training, local tech support, and caregiver-assisted sessions (49–52)
Staffing	Overburdened personnel; unclear facilitation responsibilities	Allocate dedicated facilitators; integrate with municipal roles or health volunteers (53)
Data Privacy	Low trust in confidentiality; GDPR concerns	Use localized, multilingual consent forms and clear privacy policies; ensure encryption (54–56)
Economic Viability	Reliance on short-term grants; lack of reimbursement models	Integrate telehealth into public health funding or national insurance reimbursement (57)
Transferability	Unique local factors may limit generalizability	Conduct pre-deployment readiness assessments in new regions (58, 59)

### 5.2 Digital literacy and user confidence

Digital literacy remains a critical barrier, particularly among older adults or residents with limited education. In studies across rural populations, low confidence in using health apps and medical devices has been shown to reduce willingness to engage in teleconsultations (39, 40). This challenge is not only technical but also psychological—patients may perceive remote consultations as impersonal or inadequate, fearing misdiagnosis or the absence of physical interaction (40). Such perceptions are deeply embedded in healthcare culture and must be addressed through targeted training and continuous digital support. Building digital trust requires more than training; it involves sustained user support, social proof within the community, and long-term engagement strategies.

### 5.3 Human resources and workflow integration

Although the TytoCare device is designed for guided use by non-clinicians, effective operation still depends on the availability of trained facilitators such as municipal staff, pharmacists, or community health workers. Staffing shortages in rural areas mean that existing personnel are often overburdened and lack dedicated time for new responsibilities (41). Moreover, successful telehealth requires synchronization with clinical workflows in regional hospitals. Delays in response times from remote physicians, unclear escalation protocols, or misaligned data management can reduce both effectiveness and trust in the system (42). To ensure clinical integration, escalation protocols and data handoff mechanisms must be co-designed with receiving facilities, especially in regional hospital settings.

### 5.4 Data privacy and legal concerns

Telemedicine platforms process sensitive health data, raising serious concerns about patient privacy and data security. General Data Protection Regulation (GDPR) compliance is required for any health platform operating in the EU. However, in tight-knit rural communities, social visibility is high, and residents may still fear data misuse or leakage—even when legal safeguards are in place (42). Studies show that mistrust in digital security mechanisms reduces adoption, even if legal protections are in place (43). Transparent privacy policies, local-language consent forms, and encryption guarantees are necessary to overcome these barriers (44).

### 5.5 Financial sustainability

Telehealth systems often launch with grant-based or pilot-phase funding. However, long-term sustainability is a recurrent concern. Maintenance costs, platform licensing, training updates, and broadband subscriptions all pose recurring financial demands (45). Without integration into public health budgets or insurance reimbursement schemes, the telehealth stations may suffer from operational fatigue once the pilot ends. Recent studies have shown that lack of integration into national insurance schemes or health system reimbursement plans is a major barrier to scaling telemedicine services beyond pilot phases (46).

### 5.6 Contextual transferability

The pilot's initial success is influenced by context-specific enablers—proactive municipal leadership, cohesive community networks, and existing tourism infrastructure. Caution is warranted in assuming automatic transferability to other regions without similar social or institutional readiness. Therefore, the scalability of the pilot model must be evaluated with caution, considering differences in demographics, administrative capacity, and local health system engagement. Future deployments should be preceded by readiness assessments, considering community engagement, governance capacity, and technical infrastructure. Recent findings also highlight the role of user trust and perceived usability in determining telehealth uptake in rural low-resource settings (47).

## 6 Conclusion

As a feasibility and implementation case study, the primary aim was to explore whether a scalable telehealth system could be integrated into an isolated rural community with healthcare and tourism relevance (60). The pilot implementation of a telehealth system in the remote Greek region of Agrafa presents an integrated model for advancing digital health, rural resilience, and smart tourism. Though still in an early phase, the initiative was designed to address structural barriers common to underserved rural territories—namely geographic isolation, a shortage of medical personnel, aging populations, and seasonal healthcare surges driven by tourism. The pilot also aligns with broader policy frameworks at national and European levels that emphasize digital inclusion, health equity, and sustainable regional development. This paper has outlined the multidimensional benefits anticipated from the pilot. Clinically, the intervention aims to reduce avoidable travel, improve continuity of care, and extend the diagnostic reach of overstretched healthcare systems. Socially, the deployment of community-based telehealth stations, combined with training programs, promotes digital engagement among older adults and underserved populations. Economically, the initiative may in the future enhance Agrafa's profile as a medically secure destination. The presence of tourist users during the pilot phase suggests a potential alignment with the needs of digital nomads and health-conscious travelers. Critically, the pilot offers a replicable framework for telehealth implementation in similarly situated rural regions across Europe. By positioning healthcare access as both a fundamental service and a development catalyst, the Agrafa model contributes to emerging Smart Village paradigms that integrate technological innovation with local social and economic systems. Nonetheless, several structural challenges remain. Infrastructure limitations, digital literacy gaps, human resource constraints, and data privacy concerns are not unique to Agrafa. Addressing these issues requires coordinated multi-level governance, sustained funding, and policy integration. Long-term success depends not only on technological functionality but also on ecosystem readiness—including reimbursement mechanisms, institutional alignment, and continuous user engagement. In sum, the Agrafa pilot serves as a practical demonstration of how telehealth can be embedded in rural development strategies. While empirical outcome data are forthcoming, the design and implementation approach offer important insights for future deployments. Ongoing research should focus on longitudinal impact assessment, including clinical effectiveness, community adoption, tourism engagement, and socio-economic outcomes. These findings will contribute to a growing body of knowledge on the role of digital health as a lever for inclusive rural innovation in the digital era. As rural areas seek sustainable models for healthcare delivery and territorial revitalization, telehealth may emerge not merely as a technological tool, but as a foundation for digital resilience. The Agrafa experience demonstrates that healthcare innovation, when embedded in cross-sectoral strategies, can simultaneously address social inequalities, support local economies, and reposition peripheral regions within national and European development agendas.

## Data Availability

The original contributions presented in the study are included in the article/Supplementary Material, further inquiries can be directed to the corresponding author.
